# Solvent-Free Pyrolysis Strategy for the Preparation of Biomass Carbon Dots for the Selective Detection of Fe^3+^ Ions

**DOI:** 10.3389/fchem.2022.940398

**Published:** 2022-07-06

**Authors:** Menglin Chen, Jichao Zhai, Yulong An, Yan Li, Yunwu Zheng, Hao Tian, Rui Shi, Xiahong He, Can Liu, Xu Lin

**Affiliations:** ^1^ Yunnan Key Laboratory of Wood Adhesives and Glued Products National Joint Engineering Research Center for Highly-Efficient Utilization of Forest Biomass Resources, Southwest Forestry University, Kunming, China; ^2^ Key Laboratory for Forest Resources Conservation and Utilization in the Southwest Mountains of China, Ministry of Education, Southwest Forestry University, Kunming, China; ^3^ National Joint Engineering Research Center for Highly-Efficient Utilization Technology of Forestry Resources, Kunming, China; ^4^ Agro-products Processing Research Institute, Yunnan Academy of Agricultural Sciences, Kunming, China

**Keywords:** solvent-free pyrolysis, lignin, cellulose, biomass carbon dots, metal ion detection

## Abstract

Biomass carbon dots (BCDs) have the advantages of being nontoxic, low cost and simple to prepare, have excellent optical properties, good biocompatibility and stability, and therefore have broad application prospects in areas such as heavy metal ion detection and optoelectronic devices. Herein, a simple, green, solvent-free method of preparing BCDs was developed. CDs with certain fluorescence properties were prepared by a solvent-free pyrolysis method at different temperatures using two abundant components (cellulose and lignin) of biomass resources as carbon sources. Both the cellulose CDs prepared at 300°C and the lignin CDs prepared at 350°C exhibited high quantum yields of 11.7% and 23.4%, respectively, a result that was mainly due to the high degree of graphitization. The analysis and results demonstrated the selectivity of CDs for the detection of various metal ion solutions. In particular, CDs are sensitive to Fe^3+^ and can be used as a fluorescent sensor for the detection of Fe^3+^, providing a more efficient, sustainable alternative for metal ion detection.

## Introduction

Heavy metal ions are some of the most widespread hazards that cause water pollution. The smelting, printing and dyeing industries discharge a large amount of wastewater containing heavy metal ions during rapid development, and the heavy metal ions contained in the wastewater pose a serious threat to the environment and human health when they enter the ecological environment (Gogoi et al., 2015). Currently, several methods of detecting heavy metals in water have been developed, including atomic absorption spectroscopy, inductively coupled X-ray absorption spectroscopy and surface-enhanced Raman scattering (Pyle et al., 1996; Ding et al., 2012; Sun et al., 2017). These techniques have good detection limits and have been shown to provide accurate results. The available tests have excellent detection limits and provide more accurate results for this stage of demand (Lu et al., 2017). However, they also suffer from a number of disadvantages, such as the sample preparation process is complex, the instruments used are expensive and the analysis and testing process is time-consuming and costly.

To date, many detection methods based on fluorescent nanomaterials and organic molecules have been discovered, heavy metal ions are generally detected by measuring the intensity of fluorescence signals in water bodies (Ullah et al., 2018; Ding Y. et al., 2016; Zhang et al., 2018; Xu et al., 2004). However, the syntheses of fluorescent materials and organic molecules are limited by expensive raw materials and toxic reaction solvents, which result in secondary contamination in the detection of metal ions in water bodies. Therefore, it is highly desirable to find a green, sensitive and simple method of detecting metal ions. Fe^3+^ is widespread in nature and in biological systems and is important for oxygen metabolism and electron transfer in all living organisms. Both deficiency of Fe^3+^ and excess Fe^3+^ can disrupt cellular homeostasis *in vivo*, so it is essential to detect Fe^3+^ in the environment (Chang et al., 2019; Chen et al., 2020).

As a new type of carbon nanomaterial, fluorescent CDs have attracted much attention for their unique properties, including their high-water solubility and biocompatibility (Xu et al., 2004; Liu et al., 2012). These properties enable them to provide a strong signal for the detection of metal ions in aqueous media, making them an excellent choice for environmental analysis methods. Much of the current research is focused on the properties and applications of CDs. However, the exploration of methods and raw materials for their preparation has been neglected. The common solvothermal synthesis of CDs involves expensive or toxic raw materials and solvents as well as complex processes. The search for an environmentally friendly raw material and an efficient preparation method has become a common topic of research. In terms of preparation methods, the solvent-free method is a pioneering new strategy for obtaining CDs. Hu et al. obtained CDs that can be used for metal ion detection by thermal oxidation of agricultural waste such as bitter tea residues Hu et al. (2021). Recently, Han et al. proposed a physical method of extracting CDs from carbon black Han et al. (2017). BCDs prepared from renewable, inexpensive and environmentally friendly biomass resources are also gaining interest as raw materials. In contrast to those created through conventional synthesis methods, BCDs are synthesized using only renewable natural products as raw materials, and due to the diversity of raw materials, BCDs with different properties and structures can be obtained (Zhang et al., 2016).

**FIGURE 1:**
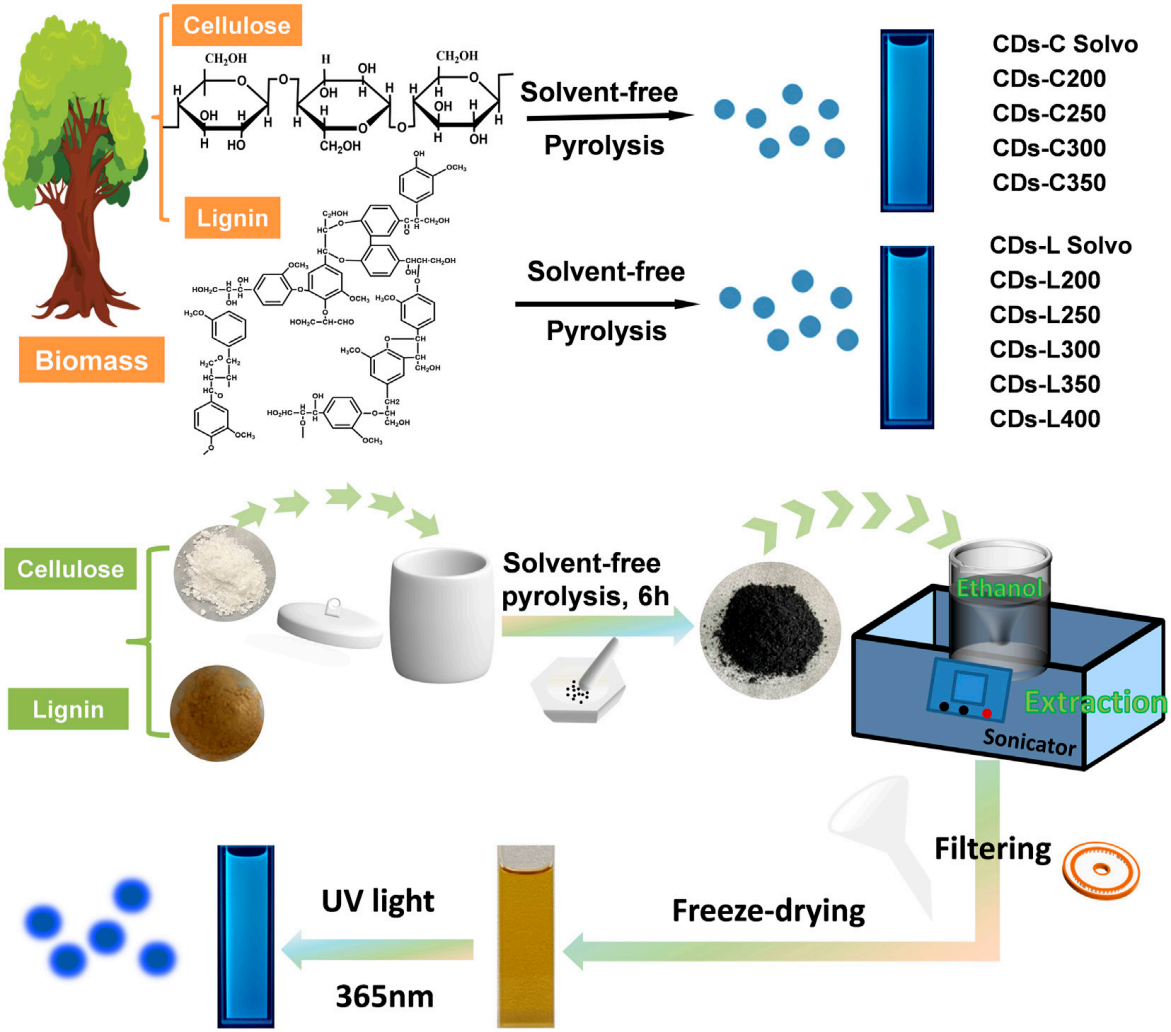
The synthesis route of cellulose- and lignin-based CDs by solvent-free pyrolysis.

Herein, we report a method for the preparation of fluorescent CDs using biomass (cellulose and lignin) by solvent-free pyrolysis. As the biomass can be incompletely charred or burnt to ash when charred at different temperatures, using 50°C as a temperature gradient, the temperature range for cellulose and lignin charcoal was 200–400°C. After biomass carbon black was obtained, it was added to ethanol, extracted for a period of time and purified to obtain a yellow carbon dot solution with bright blue fluorescence under 365 nm excited ultraviolet light (Figure 1). The CDs were named CDs-C (250–400) and CDs-L (200–400) according to the different raw materials and pyrolysis temperatures. To differential them from those prepared *via* the traditional solvothermal method, the CDs fabricated from the cellulose and lignin solvothermal method are referred to as CDs-C Solvo (180–300) and CDs-L Solvo (180–300), respectively. No blue fluorescence was observed at pyrolysis temperatures below 250°C or above 400°C for cellulose or below 200°C or above 400°C for lignin. These CDs are simple, time saving, and inexpensive to produce; the ethanol solution used for extraction is reusable, and the CDs react surprisingly well to Fe^3+^, making them strong candidates for metal ion detection.

## Materials and Methods

### Experimental Materials

Ethanol (99.7%) was provided by Shanghai Titan Science Co., Ltd. (Shanghai, China). Alkali lignin, microcrystalline cellulose were also obtained by Sinopharm Chemical Reagent Co., Ltd. Unless otherwise stated, all reagents used in this study are used as is, without further purification, and deionized (DI) water is used throughout the study.

### Preparation Process

#### Synthesis of CDs

First, 1.0 g of alkali lignin and microcrystalline cellulose were placed in separate 50 ml crucibles, heated in a muffle furnace at different temperatures for 6 h, and then cooled naturally to room temperature. The biomass-derived carbon black was ground and placed in a beaker containing 100 ml of ethanol and sonicated in a sonicator for 15 min to obtain a sample that fluoresced bright blue. This suspension is filtered to remove the carbon residue to give a solution containing carbon dots. The solution was collected carefully and then purified with a silica column chromatography using ethanol as eluents. The process was repeated three times to remove excess impurities. The final products were dried and collected under vacuum environment and dissolved in ethanol. For comparison with the solvothermal method, alkali lignin and microcrystalline cellulose (1.0 g) were dissolved in 10 ml of ethanol, and the solution was transferred to an autoclave lined with polytetrafluoroethylene. Heating in a muffle furnace at various temperatures for 6 h, cooling naturally to room temperature and filtering to remove impurities to obtain a solution containing carbon dots. The solution was collected carefully and then purified with a silica column chromatography using ethanol as eluents. The process was repeated three times to remove excess impurities. The final product was dried and collected under vacuum and dissolved in ethanol to obtain blue carbon dots for the control group.

#### Fluorescence detection of ten metal ions

In a typical assay, ten metal ion solutions (Al^3+^, Ca^2+^, Cu^2+^, Fe^3+^, Li^+^, Mg^2+^, Mn^2+^, Zn^2+^, lr^3+^, Ni^2+^) were prepared at a concentration of 1000 µM. To assess the selectivity of CDs for different metal ions, a concentration of 1000 µM of different metal ions (eg., Al^3+^, Ca^2+^, Cu^2+^, Fe^3+^, Li^+^, Mg^2+^, Mn^2+^, Zn^2+^, lr^3+^, Ni^2+^), 45ul of different metal ion solutions with specific concentrations were added to 5ul of carbon point aqueous solution and sonicated for 15 min and the fluorescence emission spectra were recorded at the same excitation light of 320 nm. A series of metal ion solutions (pH = 7.0) at concentrations of 0, 20, 50, 100, 200, 400, 600, 800, and 1000 µM were prepared in the metal ion solution (pH = 7.0) and added to the carbon dot solution in the same manner and all measurements were carried out three times.

### Characterization and Analysis

Transmission electron microscopy (TEM) images by FEI TECANI G2 F20 running at an accelerating voltage of 200 kV. UV-Visible spectra were obtained with a Shimadzu UV-2600 spectrometer. Using a Shimadzu fluorescence spectrophotometer RF-6000, the CDs fluorescence intensity was detected. Fourier transform infrared (FT-IR) spectra obtained by the KBr Pellet technique on the Nicolet iS5 spectrometer (Waltham, MA, United States) at Thermal Sciences in the primary transmission mode, and 8 scans at a resolution of 1 cm^−1^ were accumulated to obtain one spectrum. X-ray photoelectron spectroscopy (XPS) was characterised primarily by using a K-Alpha spectrometer and a single X-ray source Al Kα excitation (1486.6 eV). Binding energy calibration for C1s at 284.8 eV. Use HORIBA Scientific LabRAM HR Evolutio for Raman analysis. The sample is pyrolyzed by using the model (Japanese Science, thermo plus EV_2_/thermo mass photo) Thermogravimetry-Mass Spectrometer (TG-MS) Analyze the experiment, cellulose and lignin warming rate of 10°C/min. The quantum yields (QYs) of the CDs obtained were determined using the relative method. In particular, the use of quinine sulphate (QY = 55% in 0.1 M H_2_SO_4_) as a calibrator was chosen as a reference for the blue emission in the emission range 400–480 nm. Each experiment was performed three times in parallel to obtain the mean fluorescence measurement of QY was collected with a Shimadzu fluorescence spectrophotometer RF-6000.

## Results and Discussion

**FIGURE 2:**
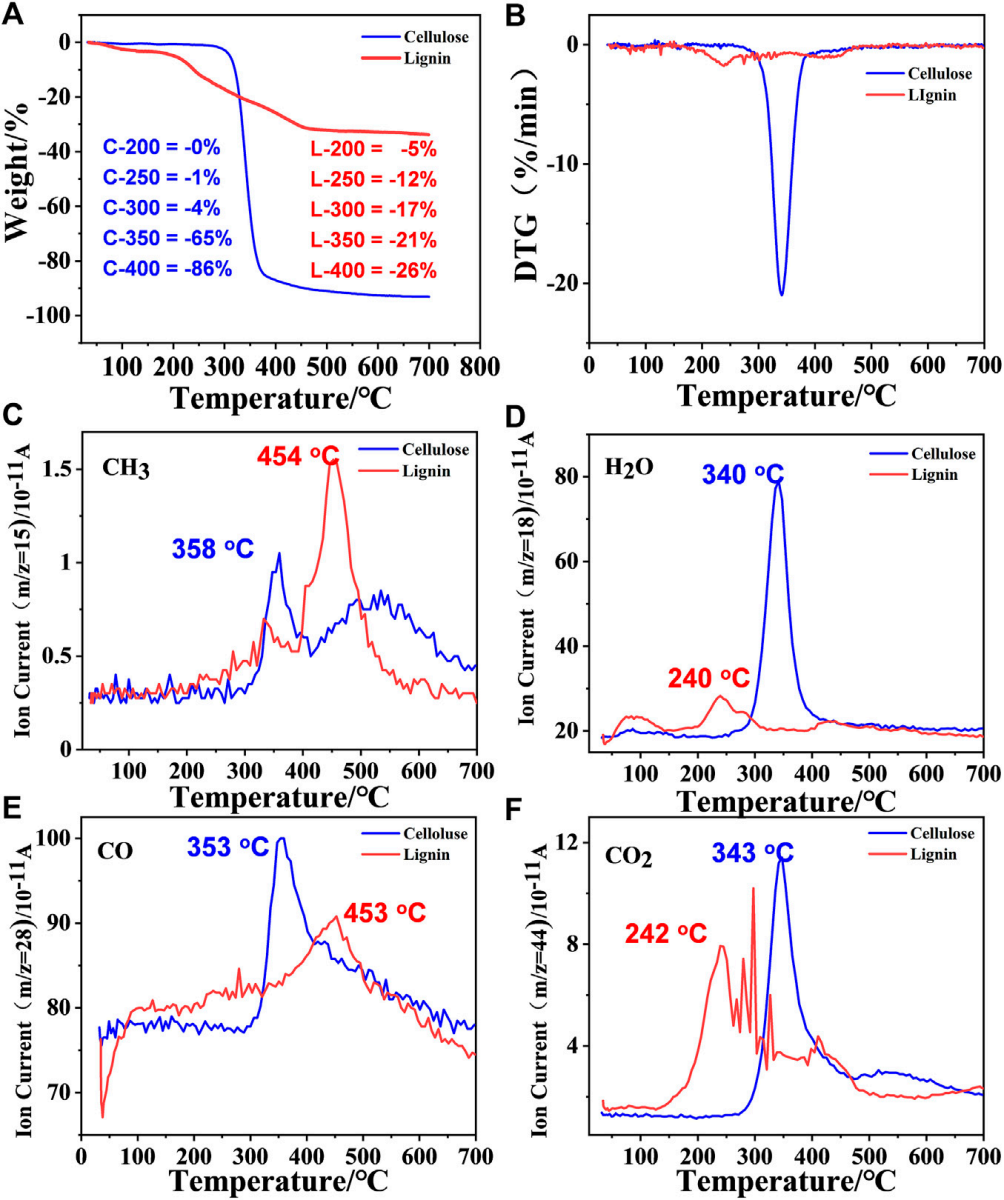
**(A,B)** TG/DTG curve of cellulose and lignin. **(C–F)** The evolutions of gaseous product for the two components at 10°C min^−1^.

The pyrolysis reactions of cellulose and lignin were compared and measured by thermogravimetric analysis and mass spectrometry (TGA-MS) (Figure 2). Both samples released weakly physisorbed water at temperatures above 100°C. As shown in Figure 2A, cellulose lose their majority of weight at 280–400°C, and the initial decomposition temperature was 250°C. The decomposition temperature range was much narrower, but the cellulose retained approximately 15% of the solid residue, which was lower than that of lignin. Compared with cellulose, lignin showed a lower initial decomposition temperature (140°C), a wider decomposition temperature range (200–500°C) and more solid residue (65%). Cellulose showed better thermal stability than lignin because it is mainly composed of carbon chain polymers connected by β-1,4 glycosidic bonds; it has regular structures, strong hydrogen bond effects and high crystallinity. Lignin, as an aromatic compound with a complex structure, has a characteristic three-dimensional network of C-C and C-O bonded phenyl propane structural units. In addition, the lignin structure is rich in hydroxyl and methoxy branched chains, their presence makes the bond energy low and prone to bond breakage (Zheng et al., 2022). Lignin has a larger number of chemical groups that require different temperatures for pyrolysis, so it requires a wider temperature range. Due to the influence of temperature, melting cyclization and the coking of aromatic structures easily occur during pyrolysis, this is one of the reasons for the large number of solid residues. The maximum weight loss rates of cellulose and lignin reached 345 and 235°C, respectively (Figure 2B).

The volatile fractions were analyzed in real time by mass spectrometry, and the four main small molecule gas products were detected at m/z = 15 (CH_3_), m/z = 18 (H_2_O), m/z = 28 (CO) and m/z = 44 (CO_2_) (m represents the number of protons, and z represents the number of charges) (Zheng et al., 2021). The elimination of the above four small molecules occurs at approximately 345°C in cellulose, which is consistent with the temperature at which the maximum weight loss rate occurs. However, for lignin, the temperatures of the four volatile components are very different. The elimination of H_2_O and CO_2_ occurs at approximately 240°C, but the elimination of CH_3_ and CO occurs at a higher temperature, approximately 450°C, which means that the formation of CDs is closely related to the elimination of H_2_O and CO_2_. The elimination of methyl groups indicates the breaking of molecular chains, which may cause the material to be less likely to form carbon dots.

**FIGURE 3:**
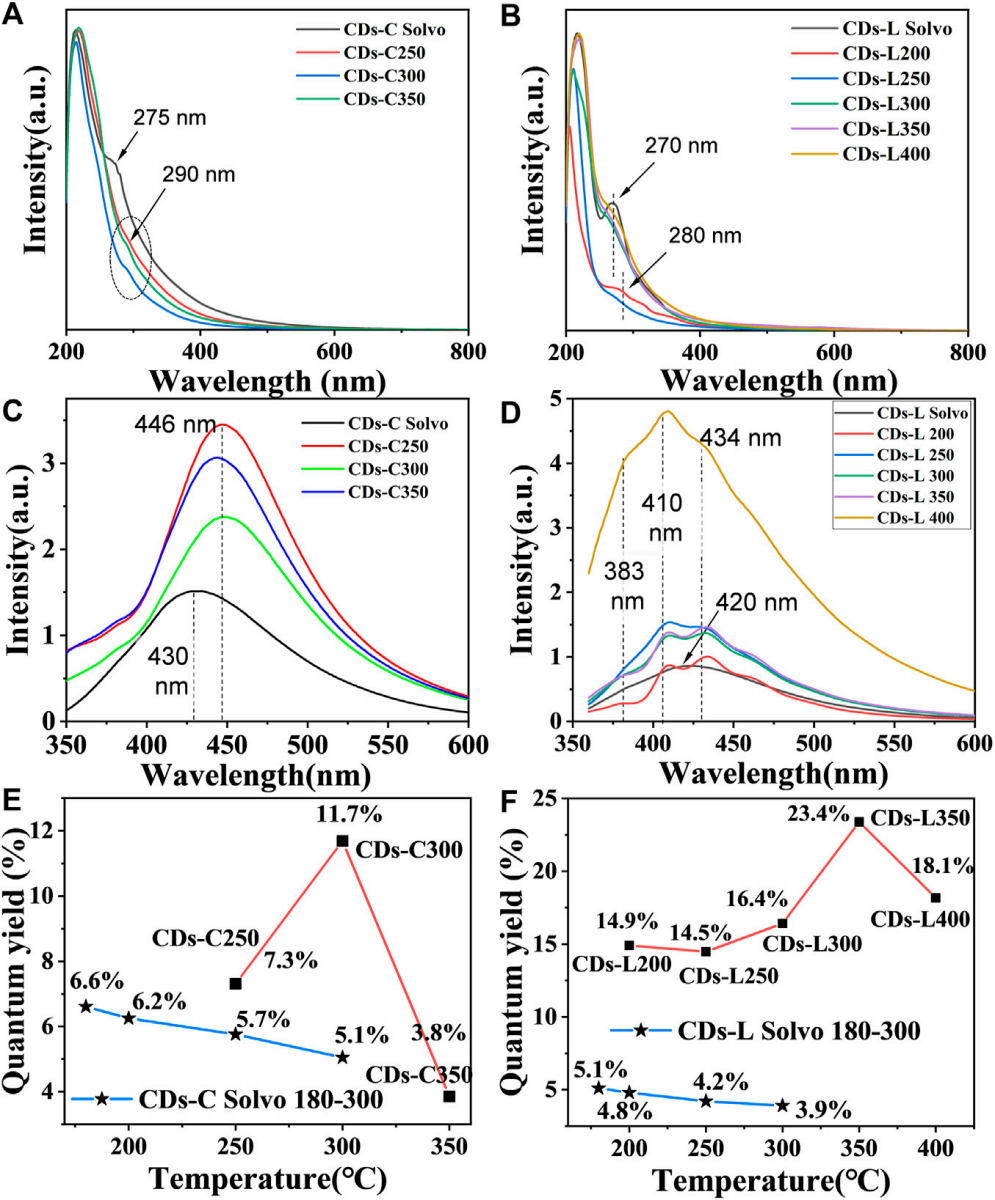
**(A,B)** UV/vis absorption spectra of CDs based on **(A)** cellulose and **(B)** lignin in ethanol solution (*c* = 1.0 mg/ml). **(C,D)** PL emission spectra of CDs based on **(C)** cellulose and **(D)** lignin in ethanol solution (*c* = 1.0 mg/ml). **(E,F)** Dotted line plot of CDs quantum yield versus reaction temperature.

The UV/Vis absorption spectra of the CDs were measured in ethanol, as shown in Figures 3A,B. In the UV spectrum, a more pronounced absorption band is shown near about 270–290 nm, which is mainly a reflection of the π-π* transition in the aromatic carbon and the n-π* transition between the sp^2^ domains. Different from many reports on CDs (Ding et al., 2017; Pan et al., 2016; Qu et al., 2012), these CDs do not show an absorption band in the UV visible region in the form of a surface defect. After varying the wavelength of the excitation light tested, no significant excitation dependence was found in the fluorescence emission (PL) spectra of the carbon dots, except for those of the CDs-L 400 obtained by carbonization at the highest temperature. The maximum fluorescence wavelength of cellulose-based CDs is approximately 446 nm, which is 16 nm redshifted compared with the CDs prepared by the solvothermal method (Figure 3C). For lignin, the CDs prepared by the two methods do not show much wavelength shift. The emission maxima of CDs-L 200–400 can be observed at λ = 383, 410, and 434 nm (Figure 3D). Different carbonization temperatures have no significant effect on the maximum emission peak of CDs but have a significant effect on the fluorescence quantum yield (QY). With increasing carbonization temperature, the QYs of these CDs first increase and then decrease. The maximum quantum yield appears at 300°C (11.7%) for cellulose and 350 °C (23.4%) for lignin. Comparing the CDs prepared by the above solvothermal method (Ge et al., 2021) revealed, that the difference in maximum fluorescence emission wavelength between the two methods was not significant at the same raw material and temperature, but the QYs of the CDs prepared by solvent-free pyrolysis were generally higher than those of the CDs prepared by solvothermal method (Figure 3E-F, Supplementary Figure S1 and Supplementary Table S1). In general, the fluorescent CDs have been prepared from cellulose and lignin by other methods such as solvothermal methods (Da Silva Souza et al., 2018, Wang et al., 2020, Xu et al., 2020). However, these methods incorporate other toxic reagents as a nitrogen source, making them complex and unsuitable for the large-scale production of carbon dots. The process of preparing biomass CDs by solvent-free pyrolysis is green and simple, and the ethanol solvent can be reused, which can broaden the application of carbon dots. The solvent-free pyrolysis method also applies to other biomass materials such as chitin and chitosan, as well as some cellulose and lignin rich biomasses such as: wood flour, bamboo flour, tree leaves and wheat straw. We found that they all have certain fluorescence properties and that the fluorescence properties varied depending on the composition of the raw material. This shows that solvent-free pyrolysis is widely applicable to most biomass materials (Supplementary Figure S2 and Supplementary Table S2).

**FIGURE 4:**
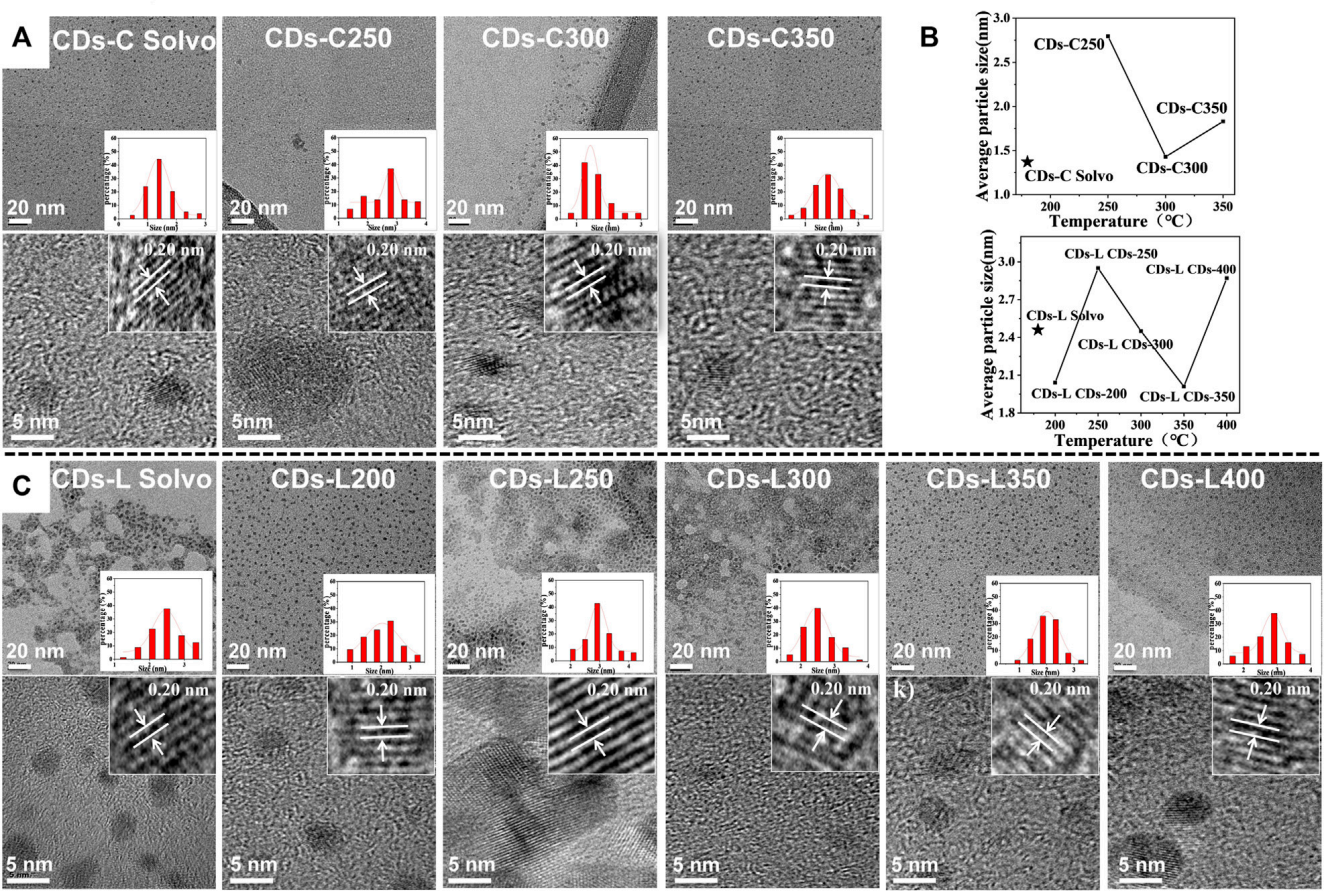
**(A)** TEM images of CDs-C Solvo and CDs-C 250–350 (from left to right), Inset: histograms and Gaussian fittings of particle size distribution, HR-TEM images of CDs-C Solvent and CDs-C 250–350 (from left to right); **(B)** TEM images of CDs-L Solvo and CDs-L 200–400 (from left to right), Inset: histograms and Gaussian fittings of particle size distribution, HR-TEM images of CDs-L Solvo and CDs-L 200–400 (from left to right); **(C)** Dotted line plot of average particle size of CDs versus reaction temperature.

Transmission electron microscopy (TEM) is an important characterization tool for examining the morphology of the resulting CDs samples. As shown in Figure 4, the TEM images show that all CDs were dot-like with uniform distribution and no obvious aggregation. The average particle sizes of these CDs in the images were found to be 1.4–2.9 nm. With increasing carbonization temperature, the particle size changed greatly, and the CDs with a high fluorescence quantum yield were the smallest. Moreover, the particle size of the lignin-based CDs was generally larger than that of the cellulose-based carbon dots, which may be related to the benzene ring structure of lignin. As shown in the high-resolution transmission electron microscopy (HR-TEM) images, the lattice stripe spacing was 0.20 nm, corresponding to the (100) crystal plane of graphite (Ge et al., 2021; Nie et al., 2014).

**FIGURE 5:**
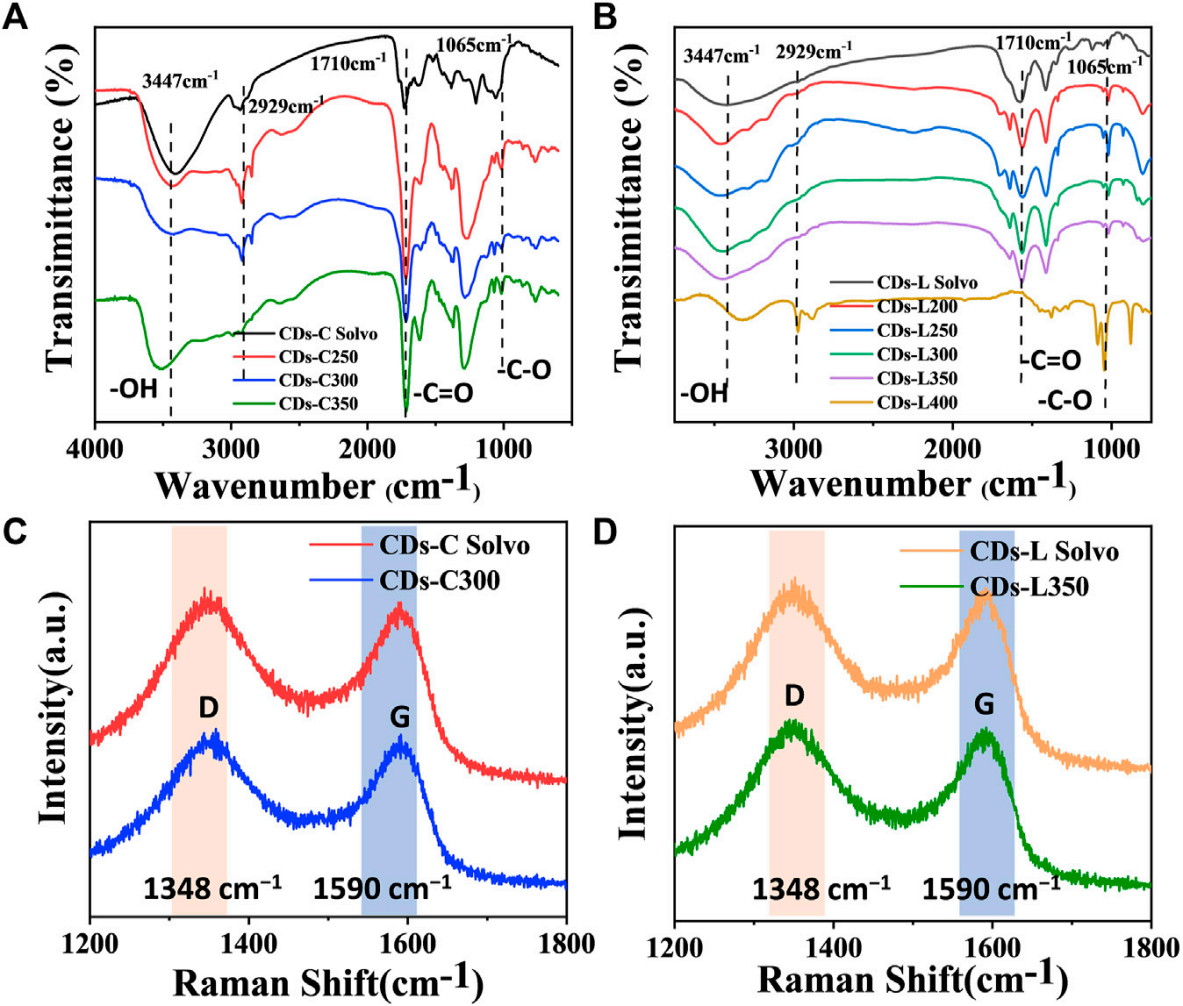
**(A,B)** FT-IR spectra of CDs based on **(A)** cellulose and **(B)** lignin. **(C,D)** Raman spectra of CDs-C Solvo, CDs-C 250, CDs-L Solvo, and CDs-L 250.

Fourier transform infrared spectroscopy (FT-IR) is an important tool for the qualitative analysis of the surface functional groups of CDs. From Figures 5A,B, these CDs show similar infrared peaks, indicating that they have similar surface functional groups and similar chemical compositions. The broad peak near 3447 cm^−1^ corresponds to the O-H stretching vibration, and it can be inferred that the presence of hydrophilic groups on the surface of CDs makes them well soluble in solvents such as water and ethanol, and that aqueous solutions of CDs are also more stable. This provides the basis for the detection of metal ions in the water column at the carbon dot. Its IR spectrum showed absorptions at 1710, and 1065 cm^−1^, which were attributed to C=O and C=C functional groups (Jiang et al., 2015). Raman spectroscopic spectra (Figures 5C,D) show two peaks at approximately 1348 and 1590 cm^−1^. According to general guidelines, the D band appears to be indicative of disordered graphite structures, whereas the G band is indicative of crystalline graphitic carbons (Wang et al., 2016; Reckmeier et al., 2016). The I_D_/I_G_ ratios of CDs-C Solvo, CDs-C 300, CDs-L Solvo and CDs-L 350 were 2.10, 2.00, 2.15, and 1.92, respectively. According to the I_D_/I_G_ ratios, when the CDs prepared from the same raw material, the graphitization of the CDs prepared by the solvent-free method was greater than that of the CDs prepared by the solvothermal method (Wang et al., 2019).

**FIGURE 6:**
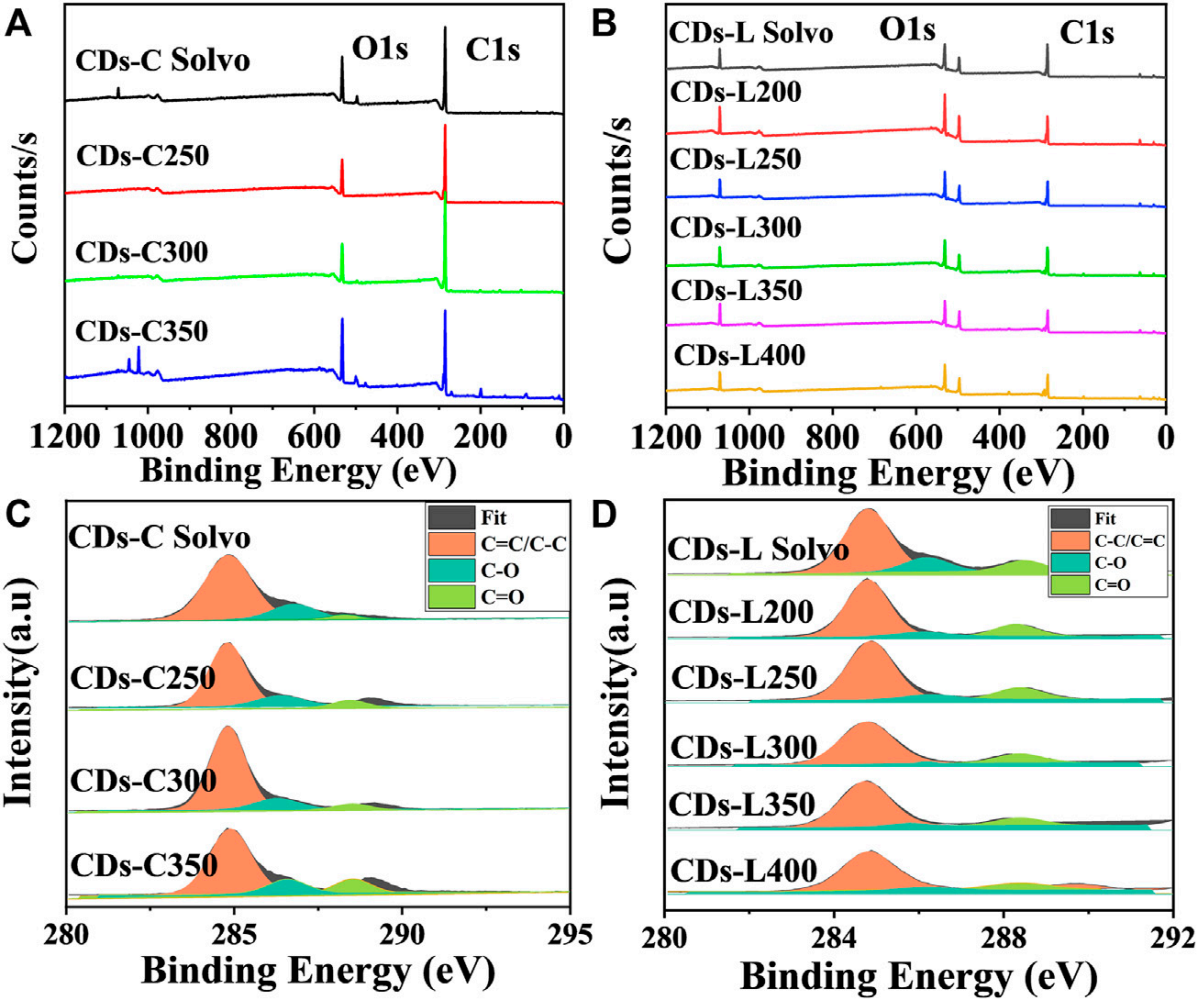
**(A,B)** XPS survey spectra, and **(C,D)** high-resolution C 1s spectra of CDs-C Solvo, CDs-C 250–350, CDs-L Solvo and CDs-L 200–400.

Further investigation of CDs surface composition is performed with X-ray photoelectron spectroscopy (XPS). As shown in Figure 6, a similar element composition was observed in all examined CDs. There are typically two intense peaks at 284.8 and 531.2 eV within the XPS spectrum, which correspond respectively to C 1s and O 1s (Sun et al., 2015; Tao et al., 2018; Yue et al., 2021). Further investigation of the surface state of the CDs, and the results are listed in Tables 1, 2. With high-resolution XPS (high-resolution X-ray photoelectron spectroscopy), surface information can be acquired for CDs. The resolved C 1s spectrum contained peaks at 284.8, 286.4, and 288.8 eV, indicating the presence of C=C/C-C (sp^2^ carbon), C-O (sp^3^ carbon) and C=O (carbonyl carbon) bonds, respectively (Hola et al., 2014; Nguyen et al., 2020). For the two kinds of CDs based on cellulose and lignin, the CDs prepared by the solvothermal method indicated that more C-O bonds were present than in those prepared by the solvent-free method, which may be attributed to the participation of solvent water in the carbonization reaction. In addition, with increasing carbonization temperature, the carbon content first increased and then decreased, which indicates that high temperatures induce the oxidative degradation of cellulose and lignin. Detailed analysis showed that the sp^2^ carbon content in CDs-C 300 and CDs-L 350 was the highest, and this was the primary factor for the high fluorescence efficiency.

**TABLE 1:** Elemental proportions and chemical bonds in cellulose based CDs.

	CDs-C solvent	CDs-C 250	CDs-C 300	CDs-C 350
C 1s	78.4	78.9%	81.7%	71.7%
O 1s	21.6	21.1%	18.3%	28.3%
C=C/C-C	77.2	75.9%	80.7%	72.4%
C-O	17.1%	15.5%	13.0%	13.2%
C=O	5.7%	8.6%	6.3%	14.4%

**TABLE 2:** Elemental proportions and chemical bonds in lignin based CDs.

	CDs-L solvent	CDs-L 200	CDs-L 250	CDs-L 300	CDs-L 350	CDs-L 400
C 1s	71.8%	70.4%	70.6%	66.2%	66.0%	56.2%
O 1s	28.2%	29.6%	29.4%	33.8%	34.0%	43.8%
C=C/C-C	72.9%	77.1%	79.1%	81.2%	83.2%	78.5%
C-O	15.8%	7.1%	5.3%	2.7%	3.1%	6.7%
C=O	11.3%	15.8%	15.6%	16.1%	13.6%	14.8%

There are two general mechanisms for the CD photoluminescence phenomenon. One is the band gap transition based on the conjugate structure in the sp^2^ carbon core, and the other is caused by a surface defect in the CDs (Ding H. et al., 2016; Ai et al., 2021). Because none of our samples indicated the absorption of surface defects, we believe that the band gap transition of the conjugated structure was the main factor controlling the PL in our system. The experimental results of Raman and XPS also support the speculation and discussion of the fluorescence mechanism. With increasing reaction temperature, cellulose and lignin condense together through dehydration and the removal of hydroxyl and other functional groups by carbon dioxide to form a conjugated graphite carbon structure and then emit fluorescence through electronic transitions. According to the experimental results, the CDs prepared by solvent-free pyrolysis have a better conjugated carbon core structure (sp^2^ carbon) and better fluorescence quantum yield than those prepared by the solvothermal method.

**FIGURE 7:**
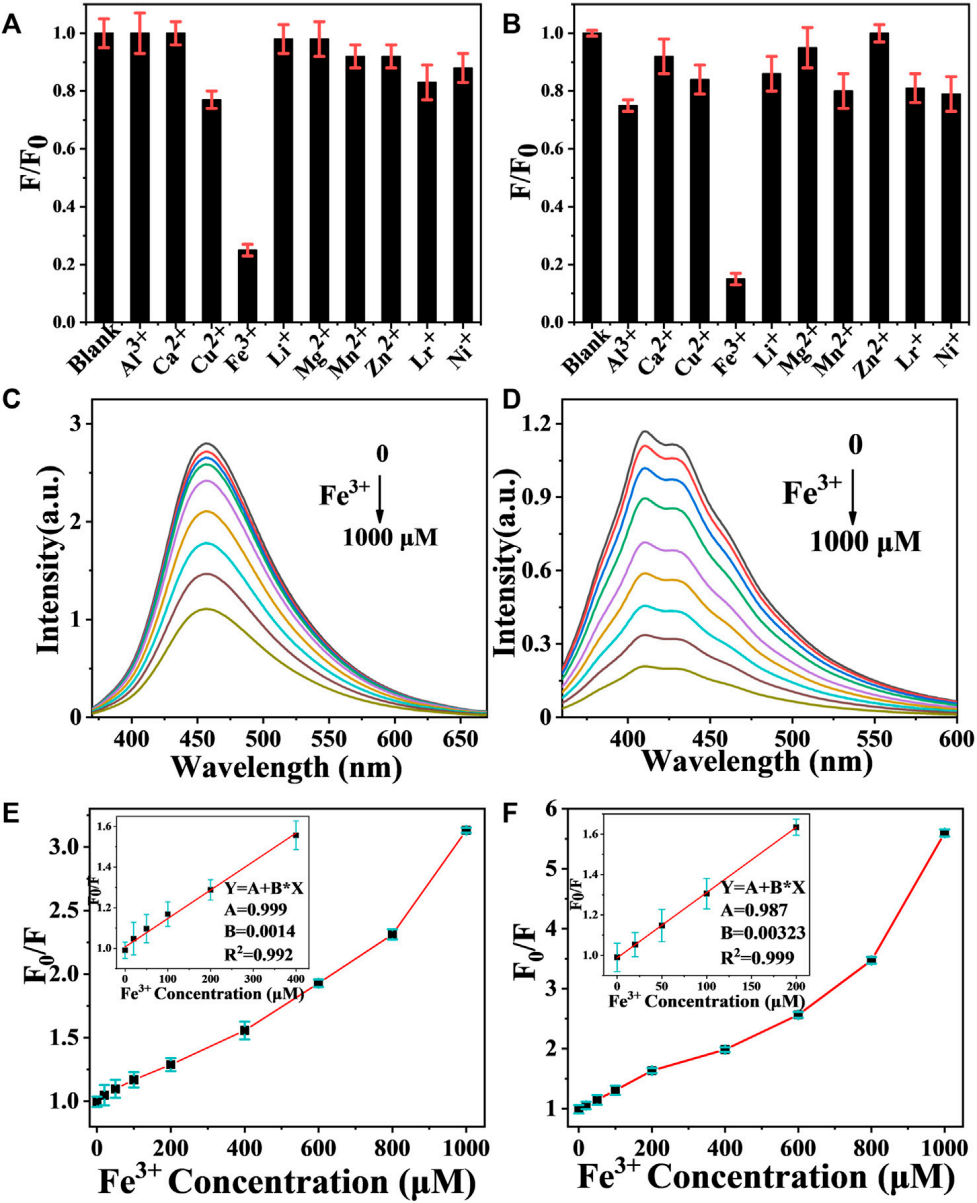
**(A,B)** Fluorescent responses of 1000 μM CDs-C 300 and CDs-L 350 to different metal ions, F_0_ and F present the fluorescence intensity of CDs before and after adding metal ions. **(C,D)** Fluorescence emission spectra of CDs-C 300 and CDs-L 350 after the addition of different Fe^3+^ concentrations. **(E,F)** Plots of the F_0_/F with different Fe^3+^ concentrations.

**FIGURE 8:**
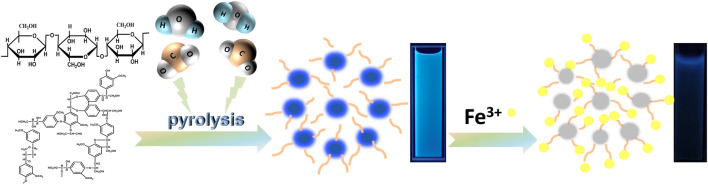
Schematic diagram of the mechanism of cellulose and lignin-based CDs synthesised by solvent-free pyrolysis for the detection of Fe^3+^.

Typically, when CDs are in the excited state, electrons are readily transferred to the metal cation, resulting in a nonradiative complex of excited state electrons and thus fluorescence quenching. Therefore, CDs is used as a fluorescent probe for the detection of metal ions in water. Compared to other common metal ion detection methods, carbon dot fluorescent probes are cost effective, easy to detect and have been extensively investigated (Meng et al., 2021). In this study, CDs-C 300 and CDs-L 350, which had the highest fluorescence efficiencies, were selected for the detection of various metal ions, as shown in Figures 7A,B. The fluorescence intensity of CDs was greatly reduced in the presence of Fe^3+^, while other ions had a weaker effect on the fluorescence intensity, indicating that CDs have excellent selectivity for Fe^3+^. We suggest that the hydroxyl group on the surface of CDs interacts with Fe^3+^, resulting in fluorescence quenching due to changes in the electronic structure of the CDs. Therefore, we further investigated the detailed sensor response of the two CDs to Fe^3+^ ions. The PL spectrum shows that the fluorescence intensity varied with Fe^3+^ concentration from 0 to 1000 μM (Figures 7C,D). Generally, the fluorescence quenching behavior of metal ions can be fitted to the Stern-Volmer equation. F_0_/F = 1 + Ksv [Fe^3+^], where Ksv denotes the quenching constant, and F_0_ and F are the fluorescence intensities before and after the addition of Fe^3+^, respectively (Bai et al., 2018). However, in our system, the plot of F_0_/F versus Fe^3+^ concentration does not conform to the conventional Stern-Volmer linear equation (Figures 7E,F). The steep upward curvature indicates that our system may involve dynamic and static quenching mechanisms in our system. Only at low Fe^3+^ concentrations (0–400 μM for cellulose and 0–200 μM for lignin), does the quenching behavior conform to the Stern-Volmer equation and exhibit a good linear relationship. The calculated Ksv values are 7.1 × 10^4^ M^−1^ and 9.3 × 10^4^ M^−1^, respectively, indicating that the two systems result from static quenching at low concentrations. The corresponding limits of detection (LODs) were 42.8 and 19.1 nM, respectively (LOD = 3δ/s, where δ is the standard deviation of 10 blank samples, and s is the slope of the linear relationship). More hydroxyl groups on the surface of the CDs lead to a negatively charged surface and more oxygen-containing groups facilitate the binding of Fe^3+^ (Figure 8). In addition, the photostability of the CDs was investigated by exposing CDs-C 300 and CDs-L 350 solutions to a 365 nm UV lamp for 7 h and to daylight for 7 days, respectively. Both were observed to be photostable above 80% (Supplementary Figures S3, S4 in the Supporting Information), indicating the long-term photostability of the probes. In addition, this study tested the thermal stability of both CDs in a water bath at 30–80°C for 3 h, and the observations showed that both exhibited good thermal stability (Yarur et al., 2019). The above experimental results indicate that CDs prepared by solvent-free pyrolysis are promising candidates for the detection of Fe^3+^ in water samples.

## Conclusion

In summary, BCDs were prepared by a solvent-free method using cellulose and lignin as carbon sources, the prepared CDs were uniformly dispersed, and all exhibited bright blue fluorescence. The synthesis temperature affected the QY and surface states of the CDs, with CDs-C300 and CDs-L350 exhibiting higher QYs of 11.7% and 23.4%, respectively, and the higher C=C/C-C content of both CDs suggests that CDs with a higher degree of graphitization can be formed at this temperature. The surface of CDs is rich in oxygen-containing functional groups, which are more conducive to the coordination of Fe^3+^ and show good sensitivity to Fe^3+^, making them suitable as fluorescent Fe^3+^ sensors. The solvent-free method of preparing CDs described in this paper is simple and green, and the reaction solvent can be reused, which not only effectively reduces the production cost of CDs but also provides a new suggestion for the efficient conversion and green development of biomass resources and a new strategy for the detection of Fe^3+^ in aqueous solutions.

## Data Availability

The original contributions presented in the study are included in the article/Supplementary Material, further inquiries can be directed to the corresponding authors.
