# Supplementary material for: Solvent-Free Pyrolysis Strategy for the Preparation of Biomass Carbon Dots for the Selective Detection of Fe3+ Ions
Source: Front Chem. 2022 Jul 6;10:940398. doi: 10.3389/fchem.2022.940398 (PMC9298851; doi:10.3389/fchem.2022.940398)
Supplement: Supplementary file 1 [file DataSheet1.docx]

**Supplementary Information**

**Solvent-Free Pyrolysis Strategy for the Preparation of Biomass Carbon Dots for the Selective Detection of Fe^3+^ Ions**

Menglin Chen^a,b^, Jichao Zhai^c^, Yulong An^c^, Yan Li^c^, Yunwu Zheng^c^, Hao Tian^d^, Rui Shi^b^, Xiahong He^b^, Can Liu^c^* and Xu Lin^a,b^ *

*^a^* *Yunnan Key Laboratory of Wood Adhesives and Glued Products National Joint Engineering Research Center for Highly-Efficient Utilization of Forest Biomass Resources, Southwest Forestry University, 300 Bailong Road, Kunming 650224, Yunnan Province, China;*

*^b^* *Key Laboratory for Forest Resources Conservation and Utilization in the Southwest Mountains of China, Ministry of Education; Southwest Forestry University, Kunming, China;*

*^c^* *National Joint Engineering Research Center for Highly-Efficient Utilization Technology of Forestry Resources, China；*

*^d^* *Agro-products Processing Research Institute, Yunnan Academy of Agricultural Sciences, Kunming, China.*

**Supporting Figures**


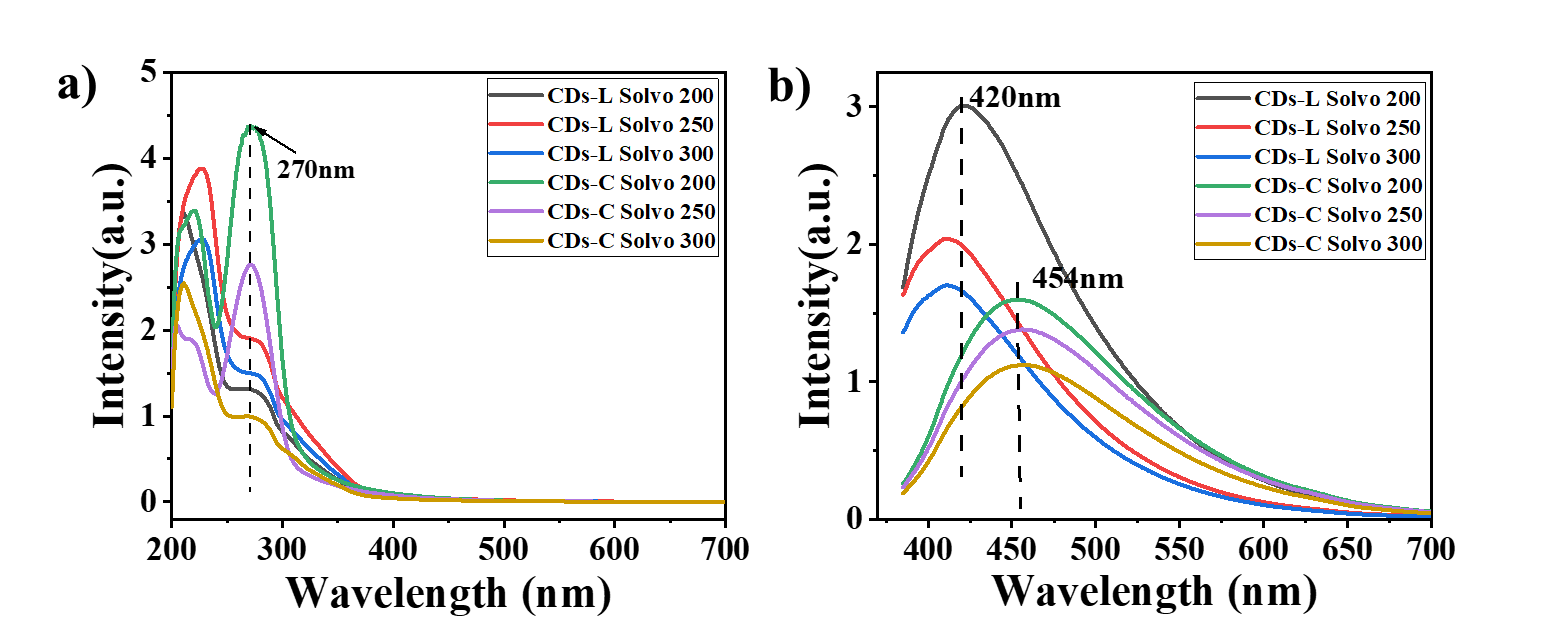


**Figure S1** UV/vis absorption spectra of CDs based on a) CDs-L Solvo 200-300 and CDs-C Solvo 200-300 in ethanol solution (*c* = 1.0 mg/mL). b) PL emission spectra of CDs based on CDs-L Solvo 200-300 and CDs-C Solvo 200-300 in ethanol solution (*c* = 1.0 mg/mL).


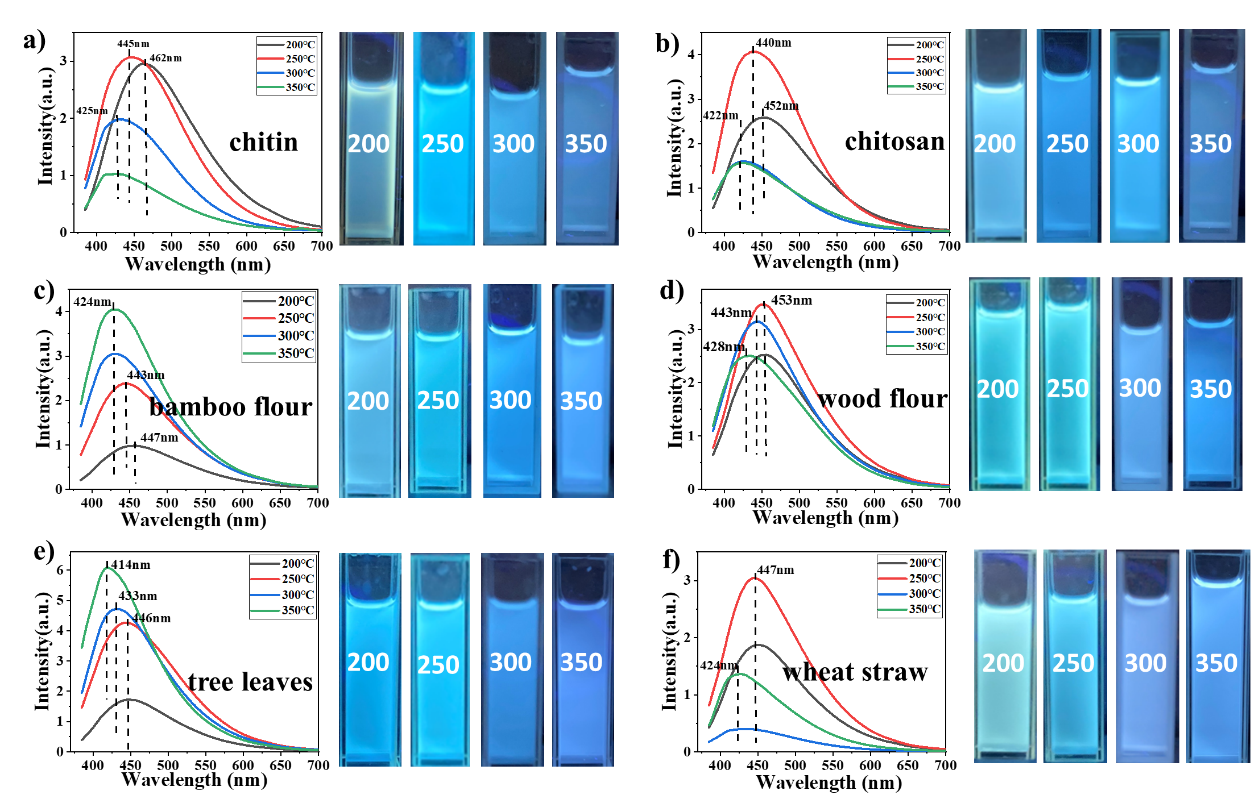


**Figure S2** PL emission spectra of CDs based on a) chitin, b) chitosan, c) wood flour, d) bamboo flour, e) tree leaves and f) wheat straw in ethanol solution (*c* = 1.0 mg/mL, λ = 365nm).


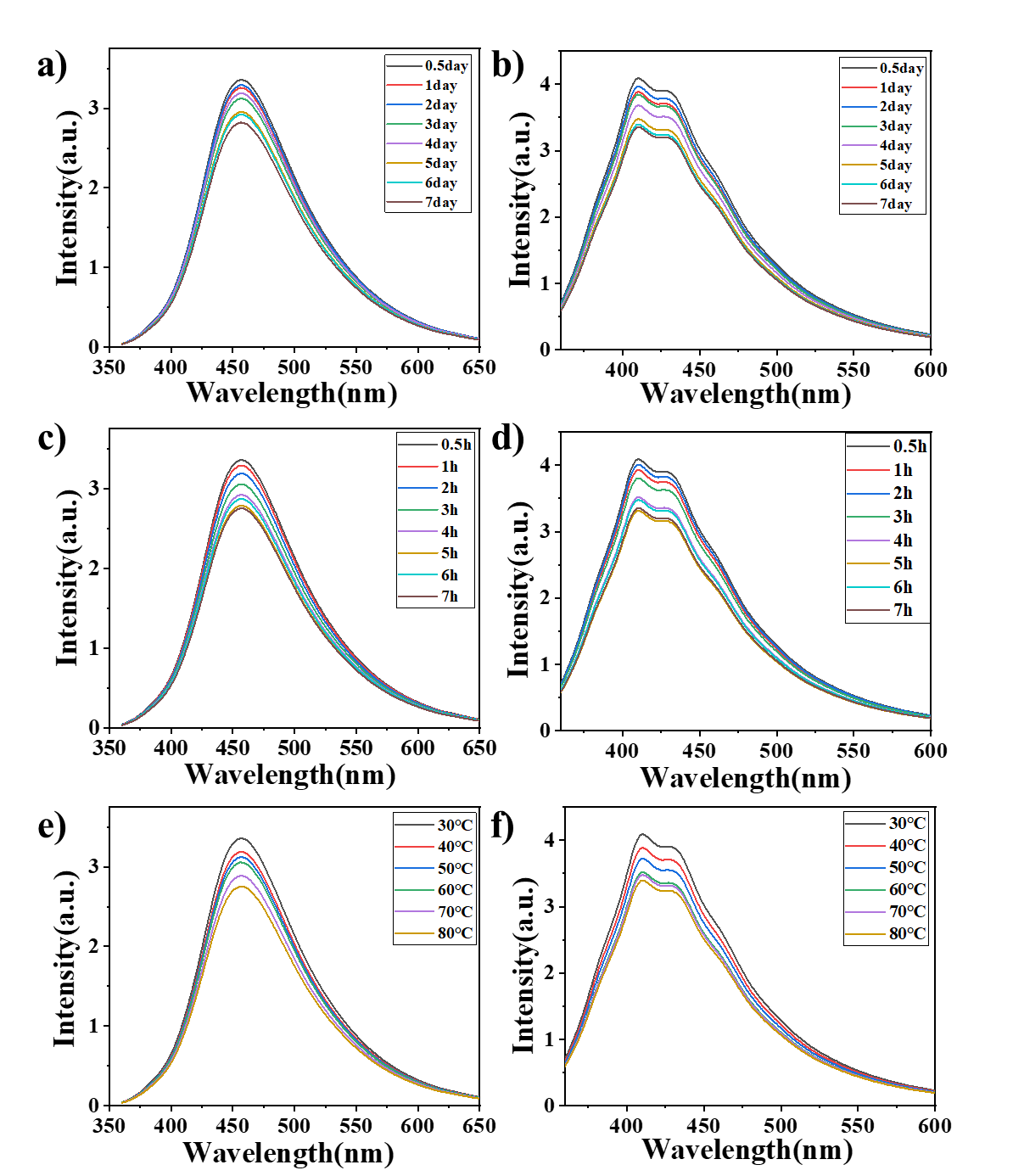


**Figure S3** a,b) PL emission spectra of CDs-C 300 and CDs-L 350 at different durations under visible light in ethanol solution; c,d) PL emission spectra of CDs-C 300 and CDs-L 350 at different durations under ultraviolet light in ethanol solution; e,f) PL emission spectra of CDs-C 300 and CDs-L 350 in a water bath at different temperatures in ethanol solution.


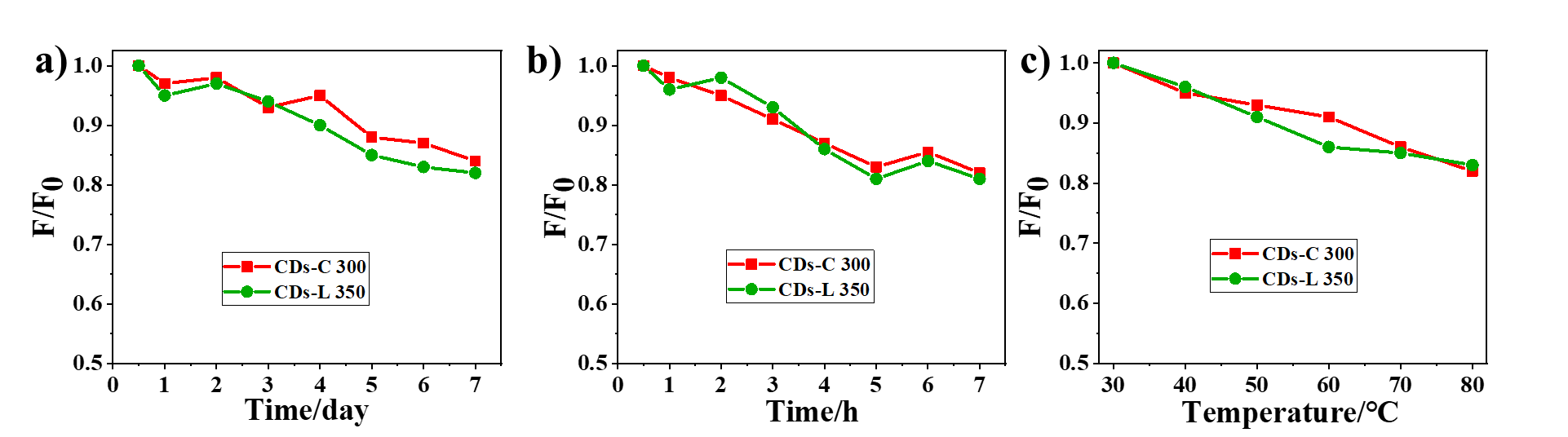


**Figure S4** Decay curve of PL intensity of CDs-C 300 and CDs-L 350 with increasing a) visible and b) UV irradiation time. c) Decay curve of PL intensity of CDs-C 300 and CDs-L 350 with increasing temperature.

**Supporting Tables**

**Table S1.** Quantum yields of CDs prepared by solvothermal method

| Name | CDs-L Solvo 200 | CDs-L Solvo 250 | CDs-L Solvo 300 | CDs-C Solvo 200 | CDs-C Solvo 250 | CDs-C Solvo 300 |
| --- | --- | --- | --- | --- | --- | --- |
| Quantum yield (%) | 8.0% | 7.3% | 6.3% | 7.1% | 6.8% | 5.4% |

**Table S2.** Quantum yields of CDs prepared from six biomass raw materials

|  | 200℃ | 250℃ | 300℃ | 350℃ |
| --- | --- | --- | --- | --- |
| chitin | 25.4% | 28.2% | 17.6% | 11.5% |
| chitosan | 27.1% | 31.8% | 21.9% | 19.3% |
| bamboo flour | 5.3% | 8.8% | 11.5 | 13.2% |
| wood flour | 15.7% | 17.2% | 14.3% | 10.4% |
| tree leaves | 7.7% | 8.6% | 10.8% | 12.5% |
| wheat straw | 9.9% | 11.8% | 5.0% | 7.5% |
